# A low-complexity compact dual-polarized patch antenna with high isolation and low cross-polarization for in-band full-duplex applications

**DOI:** 10.1038/s41598-026-45635-6

**Published:** 2026-03-28

**Authors:** Hung Tran-Huy, Trang Hoang-Thu, Tu Le-Tuan, Mohammad Alibakhshikenari, Yazeed Mohammad Qasaymeh, Takfarinas Saber, Patrizia Livreri

**Affiliations:** 1 Faculty of Electrical and Electronic Engineering, PHENIKAA School of Engineering, PHENIKAA University, Hanoi, 12116 Vietnam; 2 LERO, The Research Ireland Centre for Software, College of Science and Engineering, School of Computer Science, University of Galway, Galway, H91 TK33 Ireland; 3 Department of Electrical and Electronics Engineering, Dogus University, Umraniye, 34775 Istanbul Türkiye; 4 Department of Electrical Engineering, College of Engineering, Majmaah University, Al-Majmaah, 11952 Saudi Arabia; 5 Department of Engineering, University of Palermo, IT 90128 Palermo, Italy

**Keywords:** Engineering, Physics

## Abstract

This paper proposes a simple design of a dual-polarized microstrip patch antenna featuring compact dimensions, low cross-polarization, and high port isolation, specifically designed for full-duplex wireless applications. The antenna is implemented on a single-layer substrate, and size reduction is achieved through a capacitive loading technique. Additionally, capacitive loading also contributes to increasing polarization purity by suppressing cross-polarization radiation. A fabricated prototype with an overall size of $0.18\lambda \times 0.18\lambda \times 0.03\lambda$ at 5.6 GHz demonstrates stable performance across the frequency range of 5.53–5.62 GHz. Within this band, the antenna attains an isolation level exceeding 38 dB, a maximum broadside gain of approximately 4.3 dBi, and low cross-polarization radiation of less than $-40$ dB. Compared to other related compact dual-polarized designs, the proposed antenna exhibits the best performance in terms of isolation and cross-polarization suppression, featuring a compact and simple structure.

## Introduction

With the wireless spectrum becoming increasingly congested, the remaining available bands are highly valuable. In-band full-duplex (IBFD) or simultaneous transmit and receive (STAR) systems have emerged as promising solutions to improve spectral efficiency^1,2^. Unlike frequency- or time-division duplexing, IBFD can double spectral efficiency through transmitter–receiver cooperation. However, practical implementation requires high isolation between the transmit and receive paths, which is achieved through digital, analog, and antenna techniques^3,4^. Additionally, low-complexity design is also crucial for the ease of integration into compact, portable wireless devices.

Various antennas using microstrip patch structures with high isolation have been reported in the literature^5–9^. Such designs employ two different radiating elements, which are decoupled by external decoupling networks. Consequently, a large occupied area is required to install these antenna types. Using co-aperture dual-polarized antennas is an effective solution for compact size purposes. In^10–14^, several patches with differential feeding networks using hybrid couplers, T-junctions, or Wilkinson power dividers are proposed. These designs typically involve multi-layer structures and complex feeding networks. Although they provide wide bandwidth and high isolation, this comes at the expense of increased profile and higher structural complexity.

For improved compactness and simplicity, patch antennas with capacitive or aperture-coupled feeds offer an effective alternative. In^15–19^, patches are capacitively fed through an air gap or slots etched in the ground plane. Although high isolation is achieved, it comes at the cost of increased design complexity and antenna size. A thorough investigation of related works indicates that only a limited number of dual-polarized patch antennas featuring compact size and simple feeding schemes have been reported. In^20,21^, square patches are fed via microstrip lines or probe feeding. Other dual-polarized antennas in^22,23^ utilize fence-strip resonators or fences, allowing for compact dimensions and enhanced isolation. Alternatively, using L-shaped shorting pins and ground slots to achieve high isolation with a small footprint and low-profile structure is an advantage of the design in^24^. Overall, the current limitations of these compact dual-polarized antennas are complicated structures and/or poor inter-port isolation. Besides, they typically suffer from high cross-polarization radiation, which is just under –20 dB.

This paper presents a dual-polarized patch antenna that possesses a compact size, high isolation, and low cross-polarization radiation, all with a simple geometrical configuration. The proposed antenna utilizes a capacitive-loaded technique to achieve a compact size of $0.18\lambda \times 0.18\lambda$ at the desired operating frequency of 5.6 GHz. The proposed antenna has an operating bandwidth of 90 MHz for both dual-polarized states, ranging from 5.53 to 5.62 GHz. Across this band, the isolation is better than 38 dB, the maximum broadside gain is about 4.3 dBi, and the cross-polarization radiation is lower than $-40$ dB. Unlike conventional dual-polarized antennas that rely on multi-layer capacitive feeds, external resonators, or differential networks, the proposed work introduces an internal capacitive-loading mechanism formed by gap slots, shorting pins, and diagonal slots directly inside the patch. This integrated loading simultaneously enables size reduction, cross-polarization suppression (< 40 dB), and high isolation (>38 dB) within a simple single-layer structure, which are not achieved using existing capacitive or coupled-feed techniques.

## Single-polarized miniaturized antenna with low cross-polarization radiation

### Antenna design

**Fig. 1:**
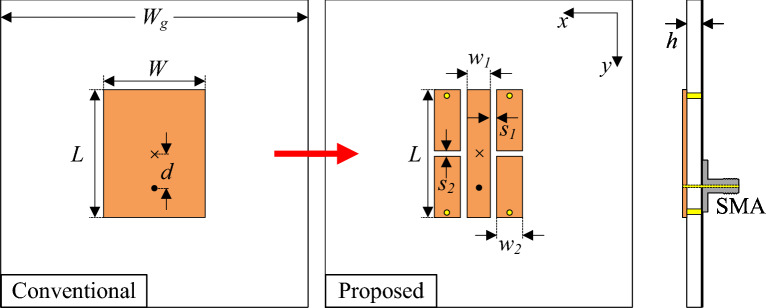
Geometry of the proposed single-polarized miniaturized antenna.

Figure 1 shows the configurations of two different microstrip patch antennas: a conventional half-wavelength patch and a capacitive-loading patch. Both antennas are printed on the top side of a Taconic RF-35 substrate, which has a dielectric constant of 3.5. For a better demonstration of the degree of miniaturization, both designs have a similar resonant length of $L = 9.8$ mm. The conventional patch antenna operates at its fundamental operating mode, TM$_{01}$. Meanwhile, the miniaturized patch is loaded with several gaps and four shorting pins. The antenna is simulated using the Ansys High Frequency Structure Simulator (HFSS) tool and the optimized dimensions of the miniaturized patch antenna is $L = 9.8$ mm, $w_1 = 1.8$, $w_2 = 2.0$, $d = 1.0$, $s_1 = 0.4$ mm, $s_2 = 0.4$ mm.

### Antenna performance

**Fig. 2:**
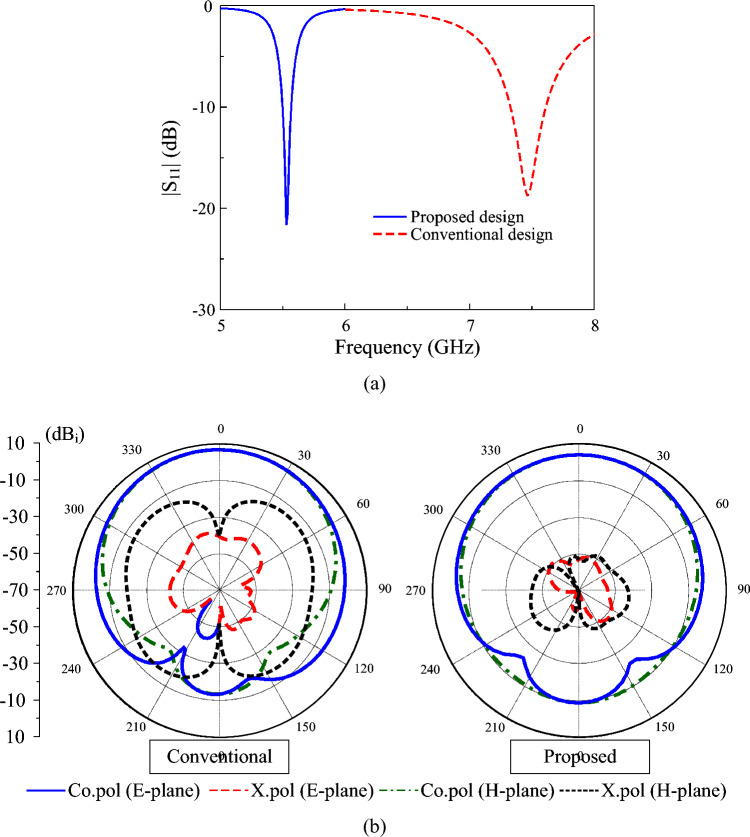
Simulated (**a**) $|S_{11}|$ and (**b**) radiation patterns of different antennas.

Figure 2 shows the simulated reflection coefficient and radiation patterns of conventional and miniaturized patches. The data in Fig. 2a indicate that the conventional patch has an operating frequency of approximately 7.5 GHz. In fact, this resonance is almost identical to the operation of the fundamental TM$_{01}$ mode. In contrast, the miniaturized patch with more coupled gaps has a lower operating frequency, around 5.55 GHz. In terms of radiation patterns, both antennas radiate good broadside beams. The conventional design has a higher gain than the proposed one, primarily due to its larger radiating aperture, which yields a gain of 6.9 dBi compared to 4.2 dBi. For the radiation pattern response, the cross-polarization radiation in the two principal planes of the miniaturized patch shown in Fig. 2b is lower than $-40$ dB. It is worth noting that this value is much smaller than that of the conventional patch, which is typically less than $-15$ dB. The reason comes from the feeding position of the proposed antenna, which is close to the patch center than the conventional design. Theoretically, the feeding position strongly affects the cross-polarization of a patch antenna because asymmetric feeding disturbs the surface current symmetry and excites unwanted orthogonal modes. The patch with smaller width results in larger impedance at the edge and then, the 50 $\Omega$ position will be closer to the patch center. Accordingly, low cross-polarization can be obtained.

It is worth noting that the low cross polarization is quite important when considering this design for dual-polarized antenna. For this antenna type, high isolation can be obtained when the coupling between the vertical polarization and the horizontal polarization is minimized, and vice versa. Thus, when the patch with vertical polarization is radiated, its extremely low cross-polarization radiation (in horizontal direction) will minimize the coupling effect to the other. Accordingly, high isolation can be obtained.

### Operation principles

**Fig. 3:**
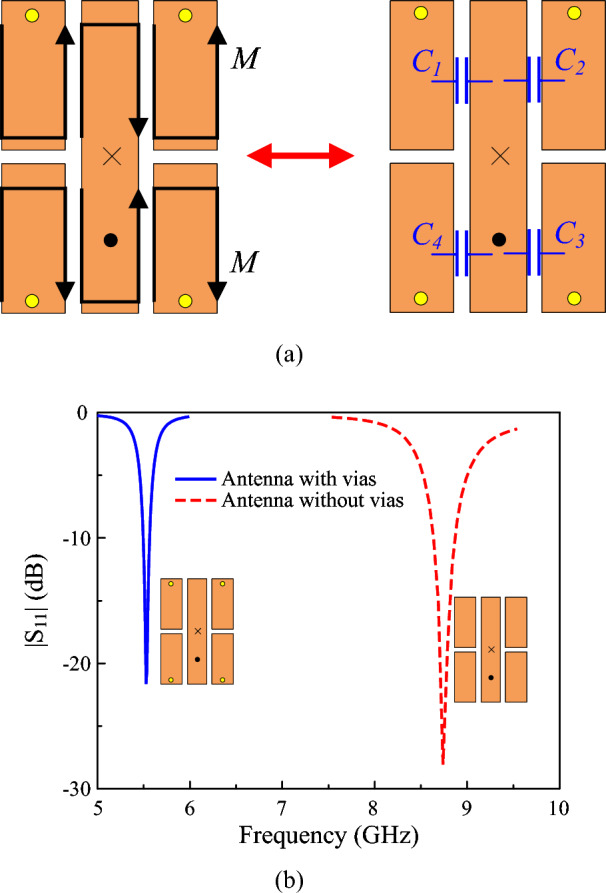
(**a**) Equivalent magnetic currents (M) and equivalent capacitive loading (C1–C4) on the miniaturized antenna structure, and (**b**) Simulated reflection coefficient ($|S_{11}|$) versus frequency for the antenna with and without shorting vias.

**Fig. 4:**
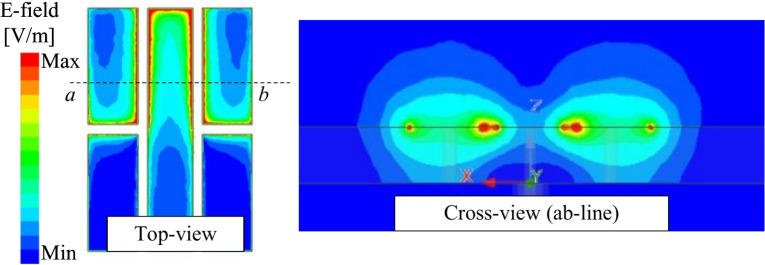
Simulated E-field distributions on the miniaturized patch.

The lower resonance could be attributed to the additional capacitance. Figure 3 shows the equivalent magnetic currents on the gap-loaded patch. It is noted that without the presence of the vias, the equivalent magnetic currents are out-of-phase. It means that the gaps will function as uncoupled gaps and thus, they have no effect on lowering the antenna operating frequency. Further investigation also indicates that the antenna without vias resonates at high frequency of around 8.8 GHz, as shown in Fig. 3b. When the vias are introduced and positioned close to the edge of the patch, the equivalent magnetic currents across the gaps are in phase. This means that there will be a strong electric field (E-field) across the gaps, and more capacitive loading will be introduced to the antenna. Accordingly, the resonance of the miniaturized antenna, which is defined by $f=1/\sqrt{LC}$, can be adjusted by controlling the additional capacitance. The simulated E-field distributions across the gaps of the miniaturized patch shown in Fig. 4 also confirm the strong coupling fields around these gaps, demonstrating the effectiveness of the utilized method in lowering operating frequency.

**Fig. 5:**
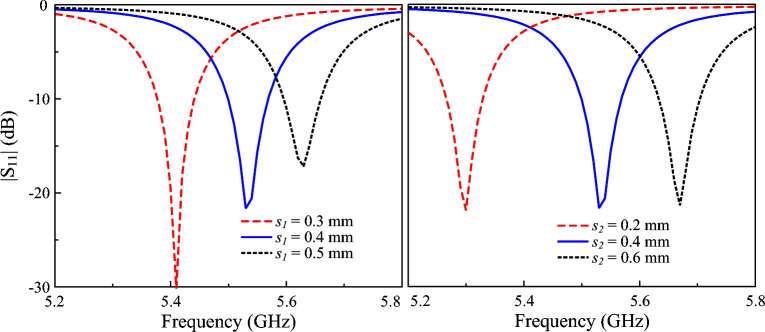
Simulated $|S_{11}|$ of the miniaturized antenna for different $s_1$ and $s_2$.

Additionally, compared with a conventional microstrip patch antenna, a capacitively loaded patch generally exhibits lower cross-polarization radiation due to improved electric-field confinement and stricter control over surface current distribution. The introduced capacitive elements effectively suppress higher-order and parasitic modes while enforcing single-mode operation, which is a critical factor for achieving low cross-polarization^25^. Figure 5 shows the simulated $|S_{11}|$ for different gap values, $s_1$ and $s_2$. The capacitance across the coupled gap is defined by $C_1 = \varepsilon A/ s_1$, in which *A* is the overlap area in proportional with the length of the coupled slot and $s_1$ is the gap of the slot. As depicted in Fig. 5a, a smaller gap results in higher capacitance, leading to lower operating frequency. Meanwhile, when changing the dimension of $s_2$, the overlapped area *A* will be changed. As bigger $s_2$ leads to smaller *A* or smaller capacitance, the resonance occurs in the higher frequency range, as shown in Fig. 5b. Further investigation also indicates that the feeding position, *d*, is a critical parameter determining the matching performance of the proposed miniaturized patch.

## Dual-polarized miniaturized antenna with low cross-polarization radiation

### Antenna design

**Fig. 6:**
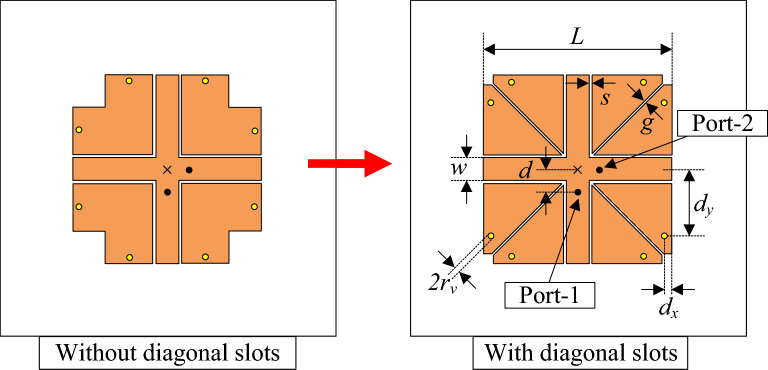
Geometry of the proposed dual-polarized miniaturized antenna.

Based on the single-polarized antenna discussed in Section II, Fig. 6 shows the geometrical configuration of the proposed dual-polarized antenna. It is worth noting that the antenna will work properly when two diagonal slots are added to the antenna. The antenna is fed at two ports, designated as Port-1 and Port-2. The optimal design parameters are $L = 9.8$ mm, $w = 1.2$ mm, $s = 0.1$ mm, $g = 0.1$ mm, $d = 1$ mm, $d_x = 0.5$ mm, $d_y = 2.9$ mm, $r_v = 0.2$ mm.

### Antenna operation

**Fig. 7:**
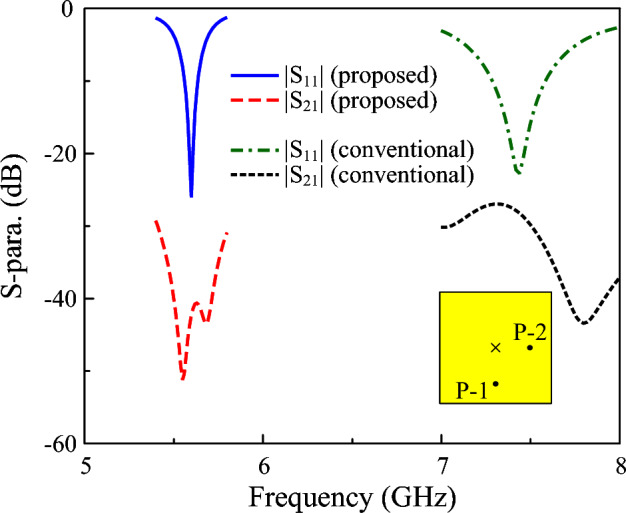
Simulated S-parameter of different dual-polarized antennas.

Firstly, the scattering parameter (S-parameter) results of the proposed dual-polarized antenna are compared to those of the conventional design. According to the data shown in Fig. 7, the conventional has an operating bandwidth from 7.29 to 7.59 GHz, and isolation is better than 27 dB. Meanwhile, despite having a narrower bandwidth (5.56–5.64 GHz), the proposed dual-polarized antenna exhibits extremely high isolation. Across the operating bandwidth, the simulated isolation values are always higher than 40 dB. The reason behind the poor isolation of the conventional dual-polarized antenna is the high cross-polarization radiation (as depicted in Fig. 2). Theoretically, the mutual coupling between two E-fields is determined by their magnitudes and the angle between them. When the fields are orthogonal, the coupling ideally becomes zero, whereas it reaches its maximum when the fields are aligned. In dual-polarized antennas, the achievable isolation is influenced by both co- and cross-polarized radiation components. Although the co-polarized fields of the two orthogonal modes (TM$_{01}$ and TM$_{10}$) exhibit minimal direct coupling due to their orthogonal orientations, significant coupling arises from the cross-polarized radiation of one mode interacting with the co-polarized radiation of the other. This occurs because these field components share the same polarization direction, thereby becoming the dominant source of mutual coupling. High cross-polarization means that the undesired polarization leaks into the desired one, increasing mutual coupling and decreasing port isolation accordingly. The simulated data demonstrates the advantages of the proposed miniaturized dual-polarized patch in comparison with the conventional one.

**Fig. 8:**
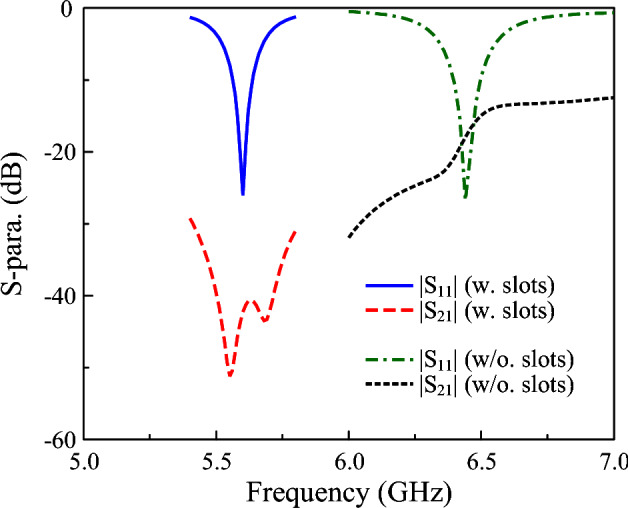
Simulated S-parameter of the proposed miniaturized dual-polarized antenna with and without diagonal slots.

Secondly, the important parameters are considered for the proposed design. As diagonal slots are embedded into the patch (shown in Fig. 6), the simulated S-parameters for the cases with and without slots are presented in Fig. 8. It can be seen clearly that the antenna without slots operates in a high frequency region around 6.5 GHz. Meanwhile, the isolation in this range is quite poor at around 20 dB. It could be attributed to the E-field coupling to undesired polarization. For the design with diagonal slots, a lower operating frequency at around 5.6 GHz can be obtained. Besides, higher isolation of better than 40 dB is also achieved. The reason for the lower resonance is that the diagonal slots will introduce more capacitance to the antenna. Meanwhile, the gap caused by the slots also reduces the coupling fields to undesired polarization.

**Fig. 9:**
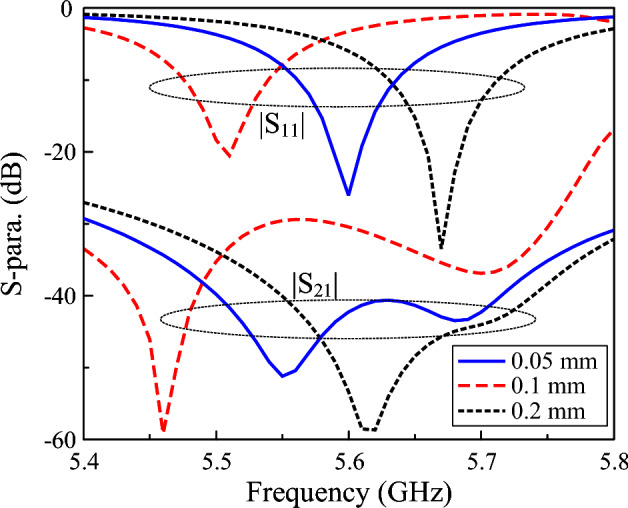
Simulated S-parameter of the proposed miniaturized dual-polarized antenna for different widths of diagonal slots.

Further demonstration can be observed in Fig. 9, which shows the S-parameter for different widths of the slot, *g*. As observed, a smaller width results in higher capacitance, and then the operating frequency shifts downwards. Additionally, a smaller width causes stronger coupling to the undesired polarization, which leads to poor isolation accordingly.

**Fig. 10:**
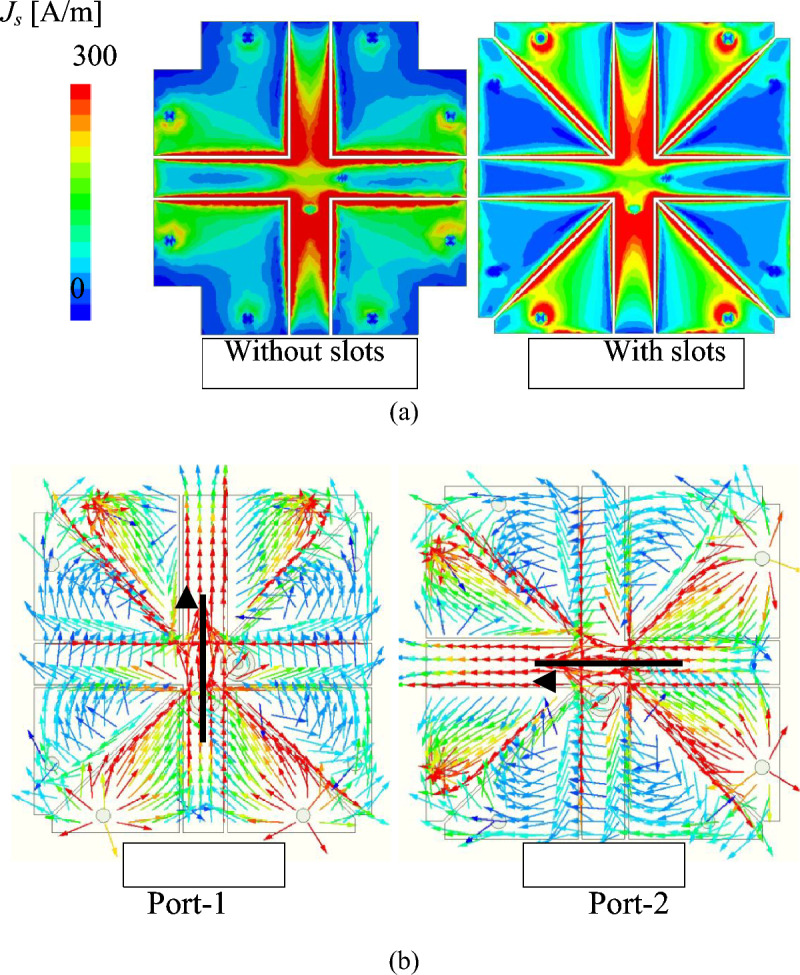
Simulated surface current distributions: (**a**) magnitude ($J_s$) for antennas with and without slots; (**b**) vector distributions for Port-1 and Port-2 showing orthogonal current paths.

Figure 10 shows the simulated surface current ($J_s$) distribution for different cases, with and without diagonal slots. It is noted that in both cases, the vertical polarization is excited. For the antenna without diagonal slots, there is a stronger current distribution on the horizontal patch. Therefore, the isolation of this design is worse than the case with diagonal slots. Additionally, it is also worth noting that the current is strongly distributed around the diagonal slots, confirming the additional capacitance of this antenna. Accordingly, lower operating frequency can be obtained.

### Measurement results

**Fig. 11:**
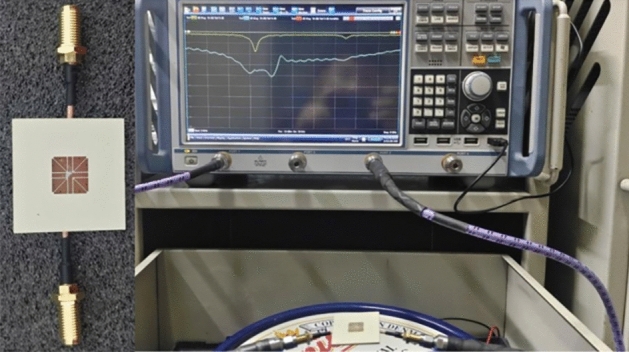
Photographs of the fabricated antenna and measurement with VNA.

For experimental validation, the fabricated dual-polarized antenna prototype was carefully characterized using a standard antenna measurement system. Figure 11 illustrates photographs of the fabricated prototype along with the corresponding measured S-parameters, which were obtained using a Vector Network Analyzer (VNA). The reflection coefficients ($|S_{11}|$ and $|S_{22}|$) and mutual coupling ($|S_{12}|/|S_{21}|$) between the two ports were measured using the VNA after performing a full two-port calibration to remove cable and connector effects.

**Fig. 12:**
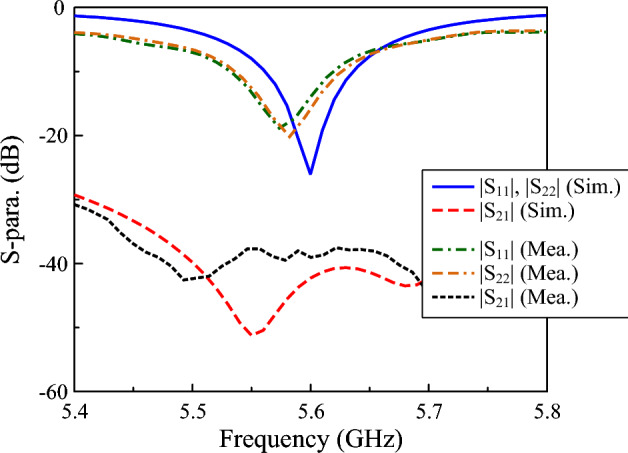
Simulated and measured the S-parameter of the proposed antenna.

Figure 12 shows the simulated and measured S-parameter of the proposed dual-polarized antenna. The measured $|S_{11}|$ and $|S_{22}|$ values below $-10$ dB across the operating band confirm good impedance matching from 5.53 to 5.62 GHz. In terms of isolation, the measured isolation between ports was observed to be greater than 38 dB, demonstrating effective decoupling between the two orthogonal polarizations. Noted that the 90 MHz bandwidth (5.53–5.62 GHz) falls within the 5 GHz Wi-Fi spectrum, where individual channels typically occupy 20 MHz, 40 MHz, or 80 MHz. Therefore, the obtained bandwidth is sufficient to support common 5 GHz Wi-Fi channel allocations. Besides, isolation levels above 35 dB are generally considered adequate for passive self-interference suppression in in-band full-duplex radios^26,27^.

**Fig. 13:**
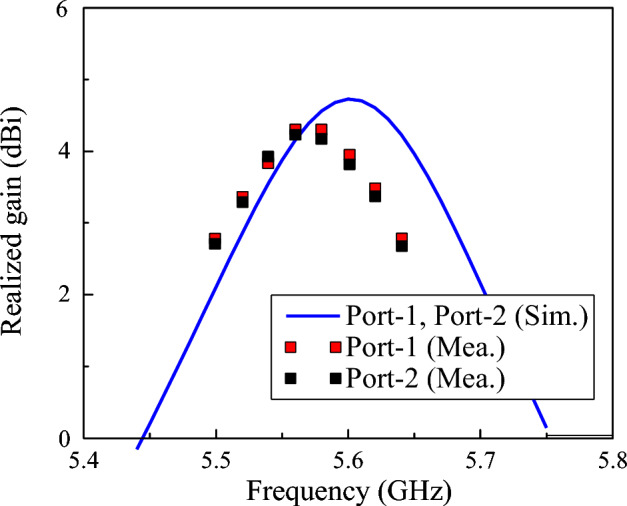
Simulated and measured gain of the proposed dual-LP antenna.

Figure 13 shows the simulated and measured broadside gain of the proposed dual-LP design. Over the operating frequency range from 5.53 to 5.62 GHz, the gain varies from 3.4 to 4.3 dBi. Additionally, the simulated radiation efficiency over this range is higher than 70%, which is acceptable for compact antenna.

**Fig. 14:**
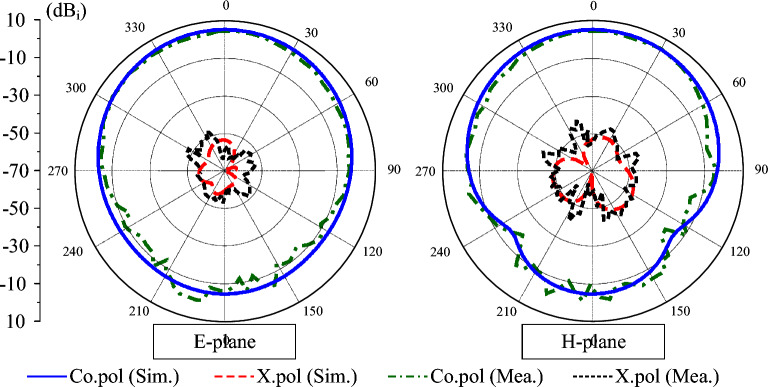
Simulated and measured gain radiation patterns of the proposed antenna.

Figure 14 plots the simulated and measured gain radiation patterns at 5.58 GHz. Owing to the similarity of the results for Port 1 and Port 2, only the radiation patterns corresponding to Port 1 excitation are presented for brevity. Both co-polarization and cross-polarization components were recorded in the principal planes (E- and H-plane) to verify the radiation characteristics and polarization purity. The measured results indicate that the antenna exhibits stable radiation patterns with low cross-polarization levels and good symmetry around the broadside direction. The measured gain is 4.3 dBi, which is slightly lower than the simulated value of 4.8 dBi. Further investigation also demonstrates that the low cross-polarization can be achieved in all planes and directions entirely the operating bandwidth.

**Fig. 15:**
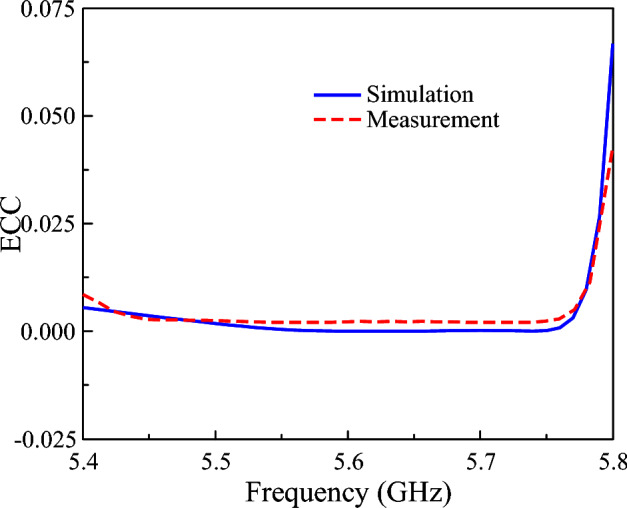
Simulated and measured ECCs of the proposed antenna.

The envelope correlation coefficient (ECC) of the proposed dual-polarized antenna is evaluated through both simulation and measurement to assess the polarization and spatial diversity performance. The simulated and measured ECC values are derived from the simulated and measured S-parameters, as expressed in Equation 1. As shown in Fig. 15, the simulated and measured ECC values show good agreement and remain below 0.05 across the operating band, confirming that the two ports are effectively uncorrelated. This low ECC demonstrates that the antenna can provide excellent diversity and MIMO performance with minimal signal correlation between the polarization channels (Table 1).

1 $$\begin{aligned} E C C_{i j}=\frac{\left| S_{i i}^* * S_{i j}+S_{j i}^* * S_{j j}\right| ^2}{\left( 1-\left| S_{i i}\right| ^2-\left| S_{j i}\right| ^2\right) \left( 1-\left| S_{j j}\right| ^2-\left| S_{i j}\right| ^2\right) } \end{aligned}$$

**Table 1:** Performance comparison among compact dual-polarized antennas.

Ref.	Size ($\lambda$)	No. of substrates	Feeding scheme	BW (%)	Isolation (dB)	Gain (dBi)	X-pol. (dB)
^15^	0.26 $\times$ 0.26 $\times$ 0.06	3	Transmission line	6.0	$\ge$ 20	8.1	$\le$ $-20$
^16^	0.20 $\times$ 0.20 $\times$ 0.24	3	Capacitive feed	2.3	$\ge$ 30	5.7	$\le$ $-20$
^21^	0.19 $\times$ 0.19 $\times$ 0.03	1	Direct	1.0	$\ge$ 28	0.9	$\le$ $-30$
^22^	0.25 $\times$ 0.25 $\times$ 0.04	2	Direct	4.9	$\ge$ 20	4.9	$\le$ $-30$
^23^	0.19 $\times$ 0.19 $\times$ 0.07	2	Direct	3.1	$\ge$ 32	4.9	$\le$ $-20$
^24^	0.16 $\times$ 0.16 $\times$ 0.04	2	Direct	3.1	$\ge$ 35	4.3	$\le$ $-20$
Prop.	0.18 $\times$ 0.18 $\times$ 0.03	1	Direct	1.6	$\ge$ 38	4.3	$\le$ $-40$

## Performance comparison

To highlight the advantages of the proposed work, a performance comparison among compact dual-polarized antennas is summarized and given in Table 1. Overall, the proposed antenna exhibits the most straightforward configuration, employing a single-layer structure with a direct feeding mechanism, while simultaneously achieving the highest isolation and lowest cross-polarization within a compact footprint. In contrast, antennas utilizing transmission-line or capacitive feeding techniques, as reported in^15,16^, require additional substrate layers, thereby increasing structural complexity and overall profile height. Comparable limitations are also observed in the designs presented in^22–24^.

## Conclusion

A dual-polarized microstrip patch antenna characterized by compact geometry, simple configuration, and high isolation has been investigated and presented in this paper. The antenna has been characterized in simulation and then validated by measurement. The final design with compact dimensions of $0.18\lambda \times 0.18\lambda \times 0.03\lambda$ at 5.6 GHz exhibits consistent performance within the operating band from 5.53 to 5.62 GHz. Across this frequency range, the impedance matching is less than $-10$ dB, the isolation is higher than 38 dB, and a peak broadside gain of about 4.3 dBi can be achieved. Compared with previously reported compact dual-polarized antennas, the proposed design demonstrates an effective trade-off among size reduction, structural simplicity, and isolation capability, making it well-suited for integration in compact modern wireless communication devices.

## Data Availability

All data supporting the findings of this study are available within the paper.
